# PDI-Spot: A Robust Representation Learning Framework for Identifying Energetic Hotspots in Protein–DNA Complexes

**DOI:** 10.1021/acs.jcim.6c01579

**Published:** 2026-10-14

**Authors:** Esra Basaran, Attila Gursoy, Ozlem Keskin, Nurcan Tuncbag

**Affiliations:** † Computational Science and Engineering Graduate Program, 52979 Koc University, Istanbul 34450, Turkey; ‡ Department of Chemical and Biological Engineering, College of Engineering, Koç University, Istanbul 34450, Turkey; § School of Medicine, Koç University, Istanbul 34450, Turkey; ∥ Department of Computer Engineering, Koç University, Istanbul 34450, Turkey

## Abstract

The binding affinity of protein–DNA complexes is concentrated within a subset of interface residues, called “hotspots”, which are critical for maintaining stable interactions. Therefore, identifying these energetic anchors is essential for elaborating on how mutations disrupt transcriptional regulation and for identifying druggable regions within disease-associated interfaces. In this study, we introduce our novel prediction model, PDI-Spot, that leverages representation learning to capture the complex features of protein–DNA interfaces. Unlike traditional methods that rely on limited, handcrafted descriptors, PDI-Spot extracts high-dimensional, structure- and sequence-driven representations to achieve better predictive performance. We trained the PDI-Spot model on a curated protein–DNA complex data set and rigorously validated its performance on an independent test set to ensure generalizability across different structural families. Following the extraction of residue-level embeddings, an optimal feature set was identified through a combined approach of minimum redundancy maximum relevance and multilayer perceptron architectures. By training a neural network on the optimal feature set, we developed a robust predictive framework for hotspot identification. On an independent test set, PDI-Spot achieved an AUROC of 0.809, an F1-score of 0.667, and a Matthews correlation coefficient of 0.625. We also verified via permutation tests that PDI-Spot captures biologically meaningful patterns beyond random chance. PDI-Spot consistently demonstrates competitive performance compared to state-of-the-art methods, providing a reliable model for mapping the energetic landscapes of protein–DNA interfaces. The source code and pretrained models are publicly available at https://github.com/ebsrn0/pdi-spot.git.

## Graphical abstract

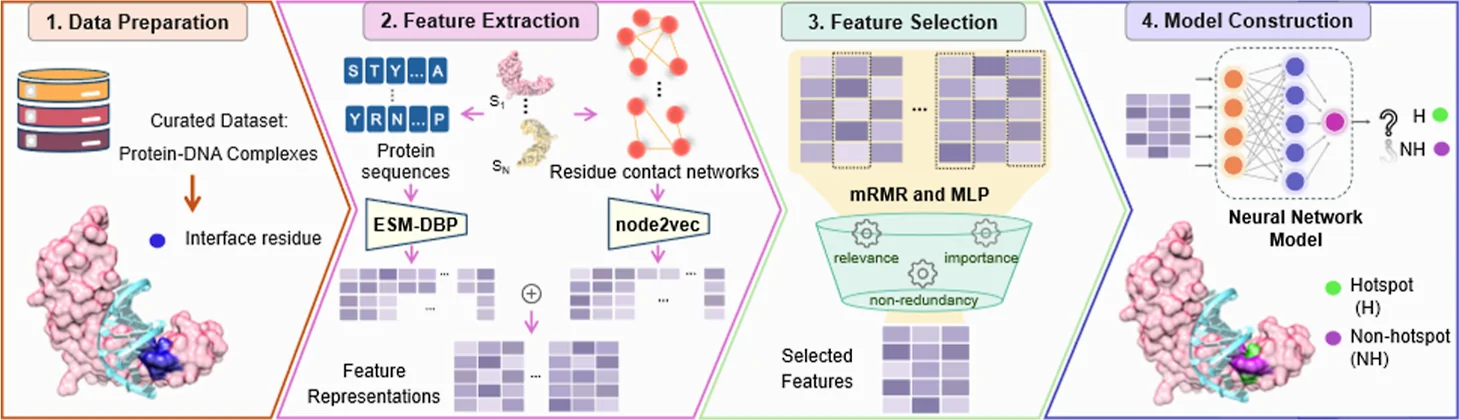

## Introduction

Protein–DNA interactions constitute the fundamental regulatory scaffold of critical cellular mechanisms such as replication, transcription, and epigenetic regulation, driven primarily by particular protein–DNA interface (PDI) residues known as hotspots that contribute to the highest binding affinity.^1–4^ Because even single-point mutations at these residues can destabilize complexes, triggering catastrophic disease-causing transcriptional failures, the precise identification of hotspots is essential for the design of targeted therapeutics and the advancement of precision medicine.^5–8^ Functionally, these critical residues are typically identified through their energetic contributions to complex stability. However, there is no strict definition of hotspots; rather, it varies based on the binding free energy change (ΔΔ*G*) upon mutation to alanine. Some methods define residues with ΔΔ*G* ≥ 1 kcal/mol as hotspots, which may yield a roughly balanced data set, while some adopt the more stringent ΔΔ*G* ≥ 2.0 kcal/mol criterion, given that hotspots represent only a small fraction of interface residues in the literature.^2,9^ These definitions have critical implications for data set construction, feature selection, model construction, and method performance comparisons with state-of-the-art methods. Although alanine scanning mutagenesis has been used to identify hotspot residues as the experimental gold standard, its low-throughput nature and dependence on high-resolution structural data cause a restrictive bottleneck for large-scale protein libraries.^10,11^ These systemic drawbacks, alongside high labor costs and significant time requirements, emphasize the necessity of robust computational methods.^12^

Computational methods for PDI hotspot prediction are broadly categorized into molecular mechanics-based and machine learning (ML)-based frameworks. Molecular mechanics methods, including SAMPDI, PremPDI, and mCSM-NA, typically quantify the change in binding free energy through force fields, graph-based signatures, or molecular dynamics simulations.^13–15^ Although they are efficient in predicting hotspots, they heavily depend on high-quality structures, thereby significantly constraining their predictive performance. Conversely, ML-based methods provide a superior alternative by leveraging the patterns within various protein features.^16^ They primarily integrate sequence, structural, and network features, followed by selecting an optimal subset. Using this feature subset, they train different ML-based algorithms, including support vector machine in PrPDH,^17^ inpPDH,^18^ SPDH,^19^ and iPNHOT;^20^ eXtreme Gradient Boosting (XGBoost) in sxPDH^21^ and SAMPDI-3D;^22^ light gradient boosting machine (LightGBM) in WTL-PDH;^12^ cat gradient boosting (CatBoost) in EC-PDH;^23^ random forest in PDH-EH;^6^ and ensemble learning in PreHots^24^ and ESPDHot.^9^ Nevertheless, important challenges remain to be overcome: the limited curated PDI data sets, class imbalance, and prevailing reliance on handcrafted features. Traditional features often fail to capture the deep latent semantics of residues, leading to less generalizable models across diverse protein families. To address this limitation, embeddings that capture intrinsic properties can be used as effective features, so that computational models are able to process and analyze them more efficiently.^25^ Although only PDH-EH utilizes embedding features, it relies exclusively on protein sequences, leaving room for improvement in performance by overlooking critical structural context. Additionally, residue network topology has previously proven to be effective in identifying clustered hotspots within protein–protein interfaces.^26^ Therefore, integrating embeddings of structurally neighboring residues can offer a promising approach for efficiently and accurately identifying hotspots at PDIs.

To address these challenges, we developed PDI-Spot, a novel model for predicting hotspot residues at PDIs by leveraging representation learning. Unlike existing methods that rely on static descriptors, PDI-Spot integrates high-dimensional embeddings from the ESM–DBP protein language model (pLM) with structural node embeddings generated via Node2Vec.^27^ By representing proteins as amino acid networks (AANs), our model captures the intricate local and global topological contexts of each residue by encoding it into a vector representation using a node embedding method, a graph-based approach that, to our knowledge, has not yet been explored in the context of protein–DNA hotspot prediction. Our model was trained and evaluated on a curated data set of 411 mutations from 139 complexes. Of these, 81 mutations from the same test set as ESPDHot,^9^ currently the top-performing method, were utilized for independent testing. PDI-Spot achieved an AUROC of 0.809 and a Matthews correlation coefficient (MCC) of 0.625 on this test set, consistently outperforming the reported performances of both ESPDHot and other state-of-the-art methods. These results demonstrate that the synergy between the pLM-derived biological context and graph-based structural topology provides a powerful foundation for predicting hotspots in PDIs. The flowchart of the PDI-Spot model is shown in Figure 1.

**Figure 1:**
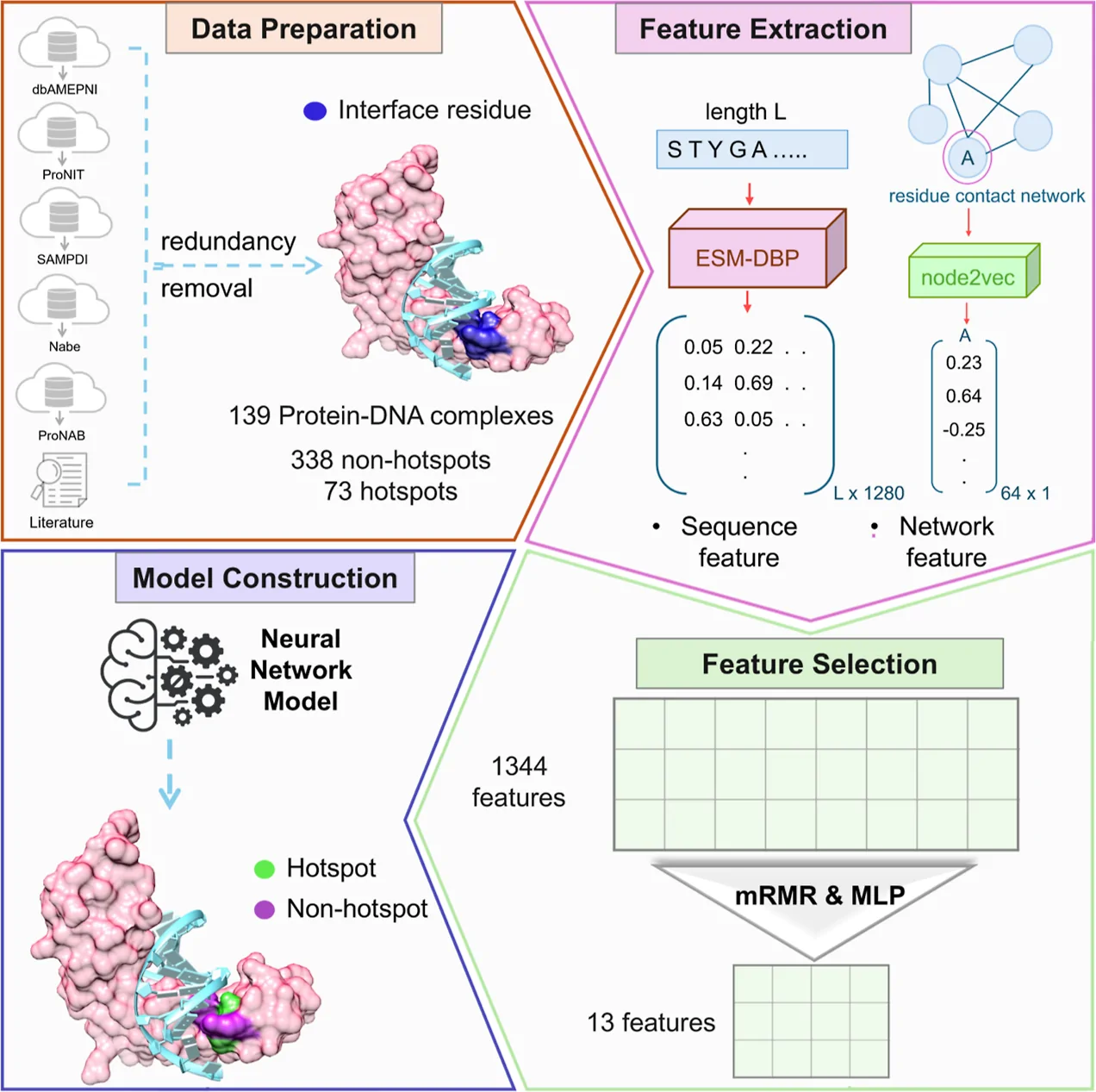
Flowchart of PDI-Spot for identifying hotspots at protein–DNA interfaces. First, the data set of 411 residue mutations from 139 protein–DNA complexes is curated. Embeddings as sequence and network features are extracted, then filtered using minimum redundancy maximum relevance (mRMR) and multilayer perceptron (MLP) to obtain a set of optimal features. A neural network model is constructed by training it as a classifier on them. Finally, the performance of our model is evaluated on the independent test data set.

## Methods

### Data Set Preparation

Two recently published data sets^9,12^ were used. The data sets of protein–DNA binding free energy changes consisted of six sources: dbAMEPNI database,^28^ ProNIT database,^29^ SAMPDI database,^22^ Nabe database,^30^ ProNAB database,^31^ and the recently published literature (10, 58, and 104 new mutation samples from the data sets used for PrPDH,^17^ PremPDI,^15^ and PreHots,^24^ respectively). To remove redundancy in the data set, a two-step filtering strategy was applied. First, CD-HIT was performed at the full-sequence level to eliminate proteins with sequence similarity >40%^32^ (Figure S2). Naccess was then performed to calculate the absolute solvent accessibility changes ΔASA.^33^ Only residues with ΔASA ≥ 1 Å^2^ after binding were retained as interface residues to exclude those that do not interact with DNA (Figure S1). Finally, the data set included 411 residue mutations (73 hotspots and 338 nonhotspots) from 139 complexes (Figure S3 and Table S1). They were divided into two sets based on a complex-based split: training set and test set, including 330 mutations (64 hotspots and 266 nonhotspots) from 113 complexes and 81 mutations (9 hotspots and 72 nonhotspots) from 26 complexes, respectively (Table S2). This split made sure that training and test sets did not share mutations from the same or homologous proteins. Notably, the test set was the same as the one in ESPDHot,^9^ thereby allowing for a fair and direct performance comparison. Here, hotspot residues were defined as those with ΔΔ*G* ≥ 2 kcal/mol, as indicated in the study by Ramos et al. This definition was used because hotspot residues play a critical role in complex formation and stability. Moreover, they are relatively few in number.^2,34,35^

### Feature Extraction

Natural language processing was performed for feature extraction. ESM–DBP^36^ was applied to generate sequence-based embeddings for DNA-binding proteins (DBPs). It gives a 1280-dimensional vector for each residue, given a protein sequence as input. Node2vec^27^ was used for network-based embeddings from AANs. It generates a 64-dimensional vector for each node, representing a residue in a protein.

#### Sequence-Based Embeddings

ESM–DBP is a method for characterization of DBPs by utilizing a domain-adaptive pretraining approach based on a pLM called Evolutionary Scale Modeling (ESM2).^36^ This model was trained on a large data set of nonredundant DBP sequences by leveraging the transformer-based architecture and self-attention mechanisms. ESM–DBP generates embeddings for residue-level annotations by capturing specific DNA-binding patterns and characteristics from protein sequences. This method generates residue embeddings using evolutionary-scale modeling. These embeddings consist of evolutionary information, DNA-binding domain characteristics—such as sequence patterns or motifs in DNA-binding domains like Homeobox or Fork-head domains—and contextual information, including the types of nearby residues.^36^ In this study, a protein sequence was given as input to ESM–DBP. For each residue, a 1280-dimensional vector was obtained as the embedding feature.

#### Network-Based Embeddings

Node2vec is a semisupervised learning method for generating feature representations of nodes in a graph.^27^ It embeds nodes into a vector space in order to map similar nodes to closer points. The method utilizes biased random walk to generate node sequences starting from each node. They are then input to a neural embedding model, the Skip-Gram model, which is trained on these sequences and maximizes the probability of predicting a node’s neighborhood given its current embedding. In this study, Node2vec was applied to AANs. To construct AANs, in which nodes represent residues and edges represent links, we followed this definition: if Cα atoms of residues are within 7 Å, edges were created. We constructed AANs for each DBP in the data set. After these networks were input into Node2vec, a 64-dimensional vector was generated for each node as the embedding feature of the corresponding residue (see Table S3 for the hyperparameters of Node2vec).

#### Handcrafted Features

Structural, physicochemical, and evolutionary features were calculated for each residue, resulting in a set of 22 handcrafted features. DSSP^37^ was utilized to extract secondary structure assignments, dihedral angles, relative solvent accessible surface area (relASA), and hydrogen-bonding features. C_α_ temperature factors (β-factors) were extracted directly from PDB structure files and subjected to Z-score normalization for each structure.^38,39^ Pairwise C_α_–C_α_ distances of H–H, H–NH, and NH–NH residue pairs were calculated as Euclidean distance from PDB coordinates, with residue pairs within 6.5 Å considered spatial neighbors. Amino acid propensity was calculated as the ratio of the frequency of amino acid among hotspot residues in the interface to its frequency in the entire protein; nonhotspot propensity was defined analogously. Hydropathy values were assigned according to the Kyte–Doolittle scale.^40^ ConSurf^41^ was used to obtain evolutionary conservation scores ranging from 1 to 9, along with exposed/buried classifications. Residues assigned grades 8 and 9 were further classified as functionally or structurally conserved based on the combination of conservation grade and solvent accessibility.^41^ These handcrafted features were used both to characterize the data set and, subsequently, to evaluate model performance in an ablation study and to assess the biological interpretability of the learned embeddings.

### Feature Selection

The higher dimensionality of features compared to the number of samples can cause an overfitting problem; the classifier may fit the training data well but fail to generalize to new samples. After concatenating sequence embeddings from ESM–DBP and network embeddings from Node2vec, this combined representation contained more than 1000 features, which is considered quite high-dimensional. Therefore, several common feature selection methods were applied exclusively to the training set to obtain nonredundant and informative features: minimum redundancy maximum relevance (mRMR),^42^ multilayer perceptron (MLP)-based selection, recursive feature elimination (RFE),^43^ and elastic net.^44^ MLP-based feature selection was performed via RFE using a single-hidden-layer MLPClassifier, in which feature importance was estimated from the aggregate input-layer weight magnitudes.^45,46^ At each iteration, the feature with the lowest importance score was removed.^47,48^ Their performance results were compared. Ultimately, we selected the intersection of features identified by both mRMR- and MLP-based selection within the training cross-validation folds as a final feature subset.

### Model Construction

Different machine learning algorithms were implemented using the AutoGluon AutoML framework^49^ with the “best_quality” preset: Random Forest, CatBoost, LightGBM, K-Nearest Neighbors, Neural Networks, and Weighted Ensemble models. The final model selected for PDI-Spot was a bagged PyTorch-based neural network model, which utilizes an MLP architecture. During the training process, AutoGluon automatically optimized the hyperparameters, and we applied balanced sample weights to mitigate class imbalance (see Table S4 for the hyperparameters of the final model).

At this step, protein-based 5-fold cross-validation was also performed to reduce the variance of prediction performance, use more data for training, and avoid overfitting.^50^ The protein-based split ensured that residue mutations from the same protein complex were restricted to the same fold, which strictly prevented data leakage during the validation. Consequently, the model was not validated on residues that were already used in the training. In addition, balancing the number of residues across the splits was considered in order to achieve an even distribution, thereby avoiding bias in model performance.

### Performance Evaluation

The performance of the model was assessed by using 5-fold cross-validation on the training set. Additionally, the model was evaluated on an independent test set to ensure its generalizability. The metrics used for performance evaluation included accuracy, recall, specificity, precision, F1 score, and the MCC. The formulas for these metrics are as follows:

$$accuracy=\frac{TP+TN}{TP+TN+FP+FN}$$

$$sensitivity\;\left(recall\right)=\frac{TP}{TP+FN}$$

$$specificity=\frac{TN}{TN+FP}$$

$$precision=\frac{TP}{TP+FP}$$

$$F1=2\times \frac{precision\times recall}{precision+recall}$$

$$MCC=\frac{TP\times TN-FP\times FN}{\sqrt{\left(TP+FP\right)\left(TP+FN\right)\left(TN+FP\right)\left(TN+FN\right)}}$$

TP stands for true positives, TN for true negatives, FP for false positives, and FN for false negatives. Furthermore, the area under the receiver operating characteristic curve (AUROC) was calculated to provide an additional measure of the model’s performance. The combination of these metrics offers a comprehensive evaluation of the model’s predictive capabilities.

Complementing these metrics, we performed two permutation-based significance analyses so as to confirm that our model’s performance was not achieved by chance. We applied a test label-shuffling and a training label-shuffling permutation test, each with 10,000 iterations. In the test label-shuffling test, class labels in the test set were randomly shuffled at each iteration with training labels kept fixed, while in the training label-shuffling test, labels in the training set were shuffled before training, generating a new randomized training set at each iteration and retraining the model on these label-shuffled sets. The distributions of the AUC, F1 score, and MCC were shown.

## Results

### Handcrafted Features Are Insufficient for Robust Hotspot Discrimination

To evaluate the discriminative power of traditional structural and evolutionary descriptors, we first characterized our curated PDI data set of 411 residues across 139 protein–DNA complexes (Table 1).

**Table 1:** Curated PDI Dataset for Model Construction for Hotspot Prediction

	protein–DNA complex	number of hotspots	number of non-hotspots	total number of mutations
training set	113	64	266	330
test set	26	9	72	81
total	139	73	338	411

Secondary structure analysis reveals that both hotspots and nonhotspots are predominantly enriched in α-helices, with significant representation of β-strands and coils (Figure 2A). The prevalence of ordered secondary structures suggests that high-affinity DNA binding is often mediated by structurally rigid frameworks. However, the presence of coils indicates that a degree of conformational flexibility is also required for effective protein–DNA interaction.^51^ To quantify this flexibility, we analyzed normalized *B*-factors, defined as the Z-scored Cα temperature factor for each PDB structure^38,39^ (see Figure S4 for a comparison with raw *B*-factors). Hotspots exhibited lower mean-normalized *B*-factors compared to nonhotspots (−0.497 vs −0.289), suggesting they reside in more stable regions (Figure 2B). However, this difference failed to reach statistical significance (*p* = 0.062), indicating that while hotspots show a preference for rigidity, atomic mobility alone is an insufficient predictor of binding importance.

**Figure 2:**
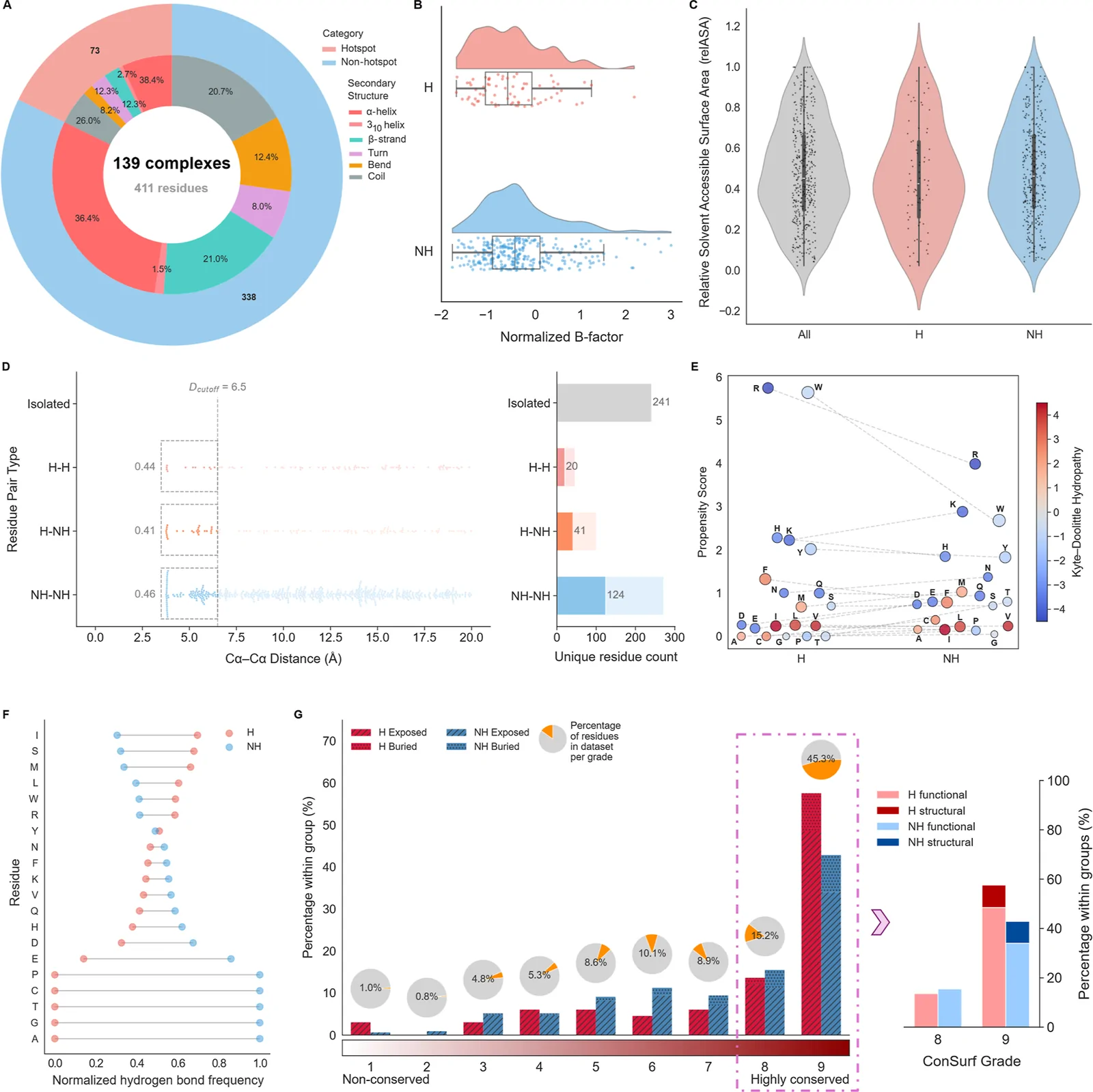
Data set is analyzed structurally and evolutionarily based on hotspot and nonhotspot residues. (A) Distribution of secondary structures across hotspot (H) and nonhotspot (NH) residues, grouped into α-helix, β-strand, turn, bend, and coil compositions. (B) Flexibility profiles based on Cα *B*-factors (values normalized within each protein). (C) Relative solvent accessible surface area for all residues, comparing the degree of exposure and burial between H and NH. (D) Spatial proximity analysis showing pairwise Cα–Cα distances for H–H, H–NH, and NH–NH residue pairs, along with the distribution of isolated residues lacking any such pairs. (E) Amino acid propensity profiles revealing which residues preferentially occur at H vs NH and how their hydrophobic properties differ between H and NH. (F) Normalized hydrogen-bond frequencies for each amino acid type, showing differences in stabilizing interactions formed by H vs NH. (G) Conservation profiles based on ConSurf grades. The distribution of exposed and buried H and NH residues is shown across ConSurf grades 1 (nonconserved) to 9 (highly conserved), along with their structural/functional conservation classification, focusing only on grades 8 and 9.

We therefore extended our analysis to solvent accessibility of hotspots and nonhotspots (Figure 2C). It is well established in the literature that hotspots in protein–protein interactions (PPIs) are more buried relative to nonhotspots.^52,53^ Our analysis found no significant difference in relASA between H and NH residues in the PDIs.

Spatial analysis of residue–residue pairwise Cα distances within PDIs further highlighted the complexity of these interfaces (Figure 2D). While the distance distribution was shown up to 20 Å, we especially focused on a 6.5 Å cutoff to identify close spatial neighbors. A small fraction of hotspot pairs (11 out of 78) fell within 6.5 Å, close enough to be in contact. We found that a fraction of hotspots (ratio of 0.44) form close spatial contacts with other hotspots, which is comparable to the fraction of nonhotspots (ratio of 0.46). Taking into account that nonhotspots highly outnumber hotspots, hotspots exhibit a considerable ratio of forming pairs. This indicates that hotspots have a distinct tendency to form clustering patterns instead of being randomly dispersed across the interface.

Physicochemical analysis identified specific residues with high hotspot propensity, notably Arginine (R) and Tryptophan (W) (Figure 2E). Arginine’s prevalence is consistent with its ability to form salt bridges and provide electrostatic complementarity with DNA. Tryptophan, additionally, contributes to π–π stacking and strong packing in the interaction.^54^ However, hydrogen-bond frequency analysis showed no clear, residue-specific trend for hotspots (Figure 2F). While R and W exhibit high H-bond frequencies, similar values are observed across various non-hotspot residue types. This suggests that while H-bonding is fundamental to the interface, it is not inherently discriminative for identifying energetic hotspots.

Finally, we investigated evolutionary conservation of hotspots using ConSurf.^41^ The majority of residues in our data set are highly conserved (grades 8 and 9), with hotspots showing a slightly higher proportion in grade 9 (Figure 2G). Notably, nearly all highly conserved residues were classified as functionally rather than structurally conserved, underscoring their direct roles in DNA-related mechanisms. Despite this trend, the high conservation of nonhotspot interface residues results in a significant signal overlap, limiting the utility of conservation scores as a standalone descriptor.

Collectively, these analyses demonstrate that while hotspots are often associated with high conservation and structural stability, no single handcrafted feature, or simple combination thereof, possesses sufficient discriminative power to accurately identify them, suggesting that the rules governing protein–DNA hotspots are encoded in high-dimensional patterns. Therefore, we propose the PDI-Spot prediction model because representation learning can capture hidden patterns that traditional descriptors overlook.

### Feature Selection Optimizes Model Predictive Performance and Model Efficiency

To optimize model performance and computational efficiency, we addressed the high-dimensional nature of our initial feature space. By concatenating sequence embeddings from ESM–DBP and network embeddings from Node2vec, we generated an initial set of 1344 features. Given that the number of features significantly exceeds the sample size, the model is susceptible to the “curse of dimensionality”, where classifiers may overfit the training data and fail to generalize to independent data sets.^55^ To mitigate this, we implemented a multistage feature selection strategy to identify a nonredundant and highly informative subset. We benchmarked several established feature selection methods, including mRMR,^42^ MLP importance, RFE,^43^ and elastic net.^44^ Evaluation of these methods on the training set (Table S5) revealed that mRMR and MLP-based selection achieved predictive metrics comparable to the other methods, while requiring a significantly smaller number of selected features than the closest comparator. To capture the top-contributing features identified by both methods, we selected 13 intersecting features as the final set, enhancing the relevance and robustness of the selected subset.^56^ This approach ensures that the selected features are not only statistically significant but also resilient to the biases of any single selection algorithm. As shown in Table 2, the intersection of mRMR and MLP features outperformed all individual and sequential selection strategies, achieving the highest F1-score (0.667) and MCC (0.625) on the test set (see also Table 3 for F1 and MCC results on both the test and cross-validation sets). To further quantify the reliability of these metrics, we calculated 95% confidence intervals (CIs) (Table S6). Ultimately, this process distilled the original 1344 dimensions into a compact, high-performance subset of 13 features, which served as the foundation for the final PDI-Spot model.

**Table 2:** Prediction Results of the Models Using Different Feature Selection Methods

feature selection methods	ACC	SEN	PRE	F1	MCC
mRMR	0.901	0.556	0.556	0.556	0.500
MLP	0.864	0.556	0.417	0.476	0.406
Intersection of mRMR and MLP-selected features	**0.926**	**0.667**	0.667	**0.667**	**0.625**
Sequential feature selection: mRMR → MLP	0.864	0.556	0.417	0.476	0.406
RFE	0.877	0.556	0.455	0.500	0.433
Elastic net	0.901	0.111	**1.000**	0.200	0.316

**Table 3:** Comparison of Cross-Validation (CV) and Independent Test Set Performance for Feature Selection Methods

feature selection methods	F1 (CV)	F1 (test)	MCC (CV)	MCC (test)
mRMR	0.44 ± 0.21	0.556	0.40 ± 0.14	0.500
MLP	0.57 ± 0.05	0.476	0.50 ± 0.06	0.406
Intersection of mRMR and MLP-selected features	0.46 ± 0.12	0.667	0.38 ± 0.11	0.625
Sequential feature selection: mRMR → MLP	0.71 ± 0.04	0.476	0.64 ± 0.07	0.406
RFE	0.14 ± 0.20	0.500	0.11 ± 0.15	0.433
Elastic net	0.79 ± 0.15	0.200	0.74 ± 0.17	0.316

To ensure that the predictive performance of PDI-Spot is not the result of random chance, we conducted two distinct permutation-based significance analyses: a test-set permutation and a training-label randomization test. Across 10,000 iterations for both tests, the model’s true performance consistently fell well outside the null distribution (*p*-value <0.01), confirming that PDI-Spot has captured genuine, nonrandom discriminative patterns within the PDI (see Figure S5 for detailed plots). To further quantify the reliability of these metrics, we calculated 95% CIs (Table S6). The range of CIs (AUROC [0.723–0.895] and MCC [0.520–0.730]) indicates that model performance exhibited predictive consistency. These results rigorously validate the robustness of our framework and demonstrate that its predictive accuracy is driven by biologically relevant features rather than statistical artifacts. However, further validation on larger independent data sets will be necessary to confirm its broader applicability.

### PDI-Spot Shows Competitive Performance against the State-of-the-Art Methods for the PDI Hotspot Prediction Task

To evaluate the robustness and comparative performance of PDI-Spot, we conducted a benchmark analysis against several established PDI prediction methods, including both classification-based (PreHots, PrPDH, and ESPDHot) and regression-based (mCSM-NA, SAMPDI-3D, and premPDI) models, using their performance reported on the test set in the ESPDHot study.^9^ As summarized in Table 4, PDI-Spot demonstrated superior performance across nearly all evaluation metrics. Notably, our model achieved the highest MCC (0.625) and F1-score (0.667), significantly outperforming ESPDHot (MCC: 0.516, F1: 0.571) and other existing state-of-the-art tools (see Table S7 for a comprehensive performance comparison). While several regression-based models exhibited high specificity, they often suffered from extremely low sensitivity, failing to identify a meaningful number of true hotspots. In contrast, PDI-Spot maintains an optimal balance between precision and recall. These results underscore the effectiveness of combining sequence and network embeddings with feature selection strategies. Collectively, this comparative analysis shows that PDI-Spot has promising robust predictive capability on the independent test set for the identification of energetic hotspots at PDIs. To further verify that the better performance is not attributable only to the larger training set used in PDI-Spot, we retrained PDI-Spot on the training data of ESPDHot and evaluated it on the same test set. PDI-Spot still outperformed ESPDHot under this fair comparison (Table S8).

**Table 4:** Comparison of Our Method with ESPDHot on the Test Set

methods	ACC	SEN	SPE	PRE	F1	MCC
ESPDHot	0.889	**0.667**	0.917	0.500	0.571	0.516
PDI-spot	**0.926**	**0.667**	**0.958**	**0.667**	**0.667**	**0.625**
PDI-spot (static descriptors)	0.779	0	0.870	0	0	–0.124

### Nonoverlapping and Complementary Sequence and Network Features Are Critical for Accurate Prediction

Through the intersection of the mRMR and MLP selection, we identified an optimal subset of 13 features. Permutation-importance analysis using AutoGluon, which quantified their individual contributions, indicated that while sequence-derived embeddings provided the primary predictive contribution, network-derived embeddings contributed a necessary layer of discriminative power that sequence alone cannot capture (Figure 3A). Furthermore, since minimum redundancy and maximum relevance in the feature set are important for a robust predictive model,^42^ pairwise Pearson correlation analysis was performed on the selected feature set. This analysis confirmed minimal multicollinearity, indicating that these features are complementary and capture distinct, nonoverlapping aspects of PDIs (Figure 3B).

**Figure 3:**
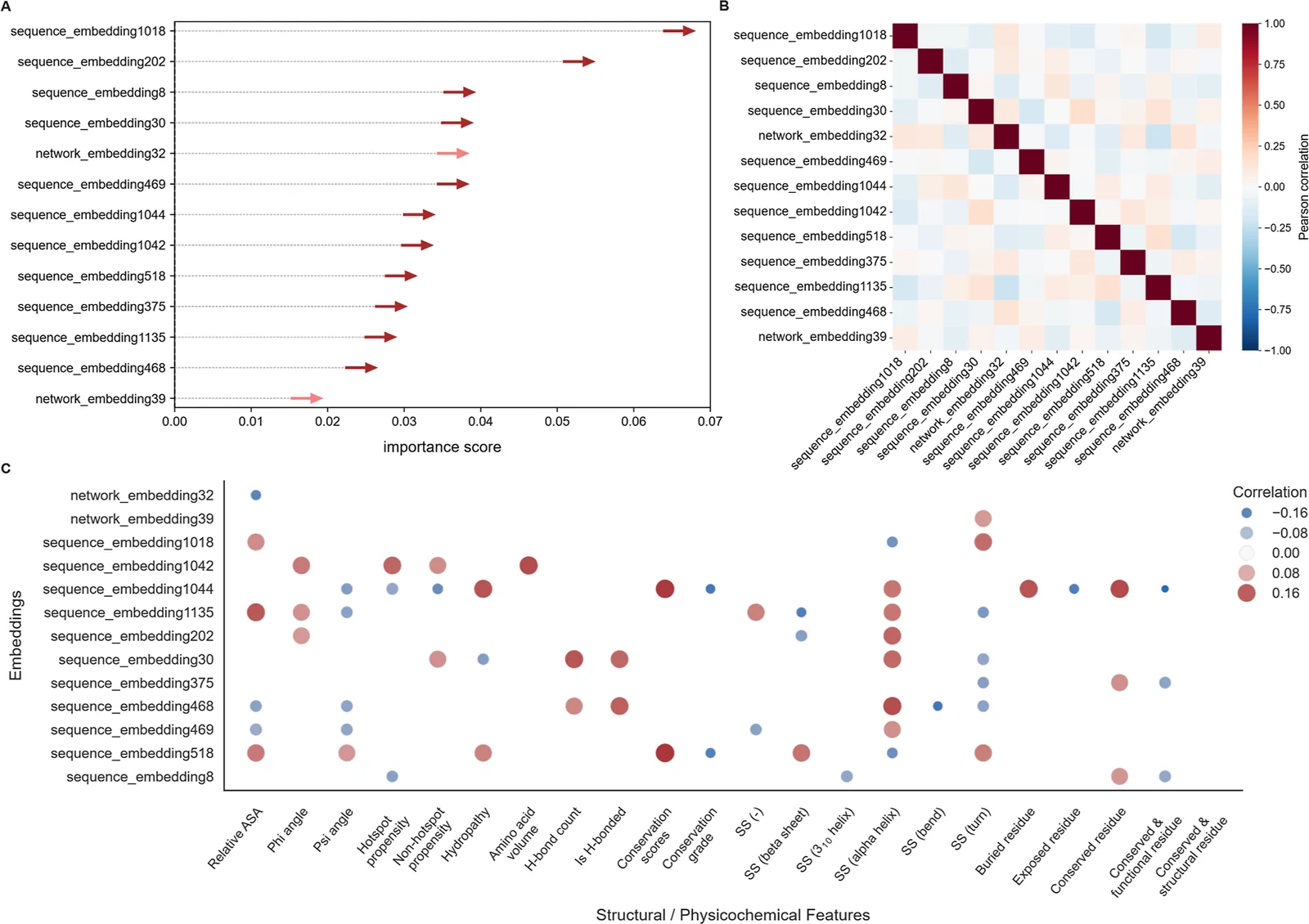
(A) The feature importance of the selected 13 features. Each rightward-pointing arrow illustrates a feature’s positive contribution to the model, with its importance score indicated on the *x*-axis. Dark red arrows represent sequence-based embeddings, while light red arrows show network-based embeddings. (B) Pearson correlation heatmap showing the pairwise correlations among these 13 important features. (C) Correlation between learned embeddings and classical structural descriptors. Spearman correlation analysis between representative dimensions of the learned embeddings (*y*-axis) and conventional handcrafted features (*x*-axis). SS on the *x*-axis refers to the secondary structure used in DSSP, which labels it as categorical variables such as SS_helix and SS_turn. Dot size and color intensity represent the magnitude of the correlation coefficient, with blue and red indicating negative and positive correlations, respectively.

Finally, we conducted an ablation analysis to evaluate the synergy between feature types (Table 5). Models trained exclusively on sequence features exhibited high training performance but a substantial generalization gap, as evidenced by the large differences in F1-score and AUC between the training and test sets (ΔF1 = 0.37 and ΔAUC = 0.29). In contrast, the model trained on network features exhibited insufficient discriminative power. However, the integration of both feature types resulted in a compelling synergistic effect; the combined model achieved the highest test performance while significantly narrowing the generalization gap (ΔF1 = 0.19 and ΔAUC = 0.17). These results demonstrate that combining sequence and network embeddings significantly contributes to building a robust predictive model for hotspot prediction. To further validate these findings, we extended the ablation analysis to include simpler baseline models and alternative feature configurations. As summarized in Table S9, no single feature type or simpler model architecture consistently matched PDI-Spot’s performance, confirming that the observed improvements arise from the synergy between representation learning, the proposed feature-selection strategy, and AutoGluon-optimized model training.

**Table 5:** Performance Comparison of Sequence and Network-Based Features on Train and Test Sets

features		ACC	SEN	PRE	F1	MCC	AUC	ΔF1 (train-test)	ΔAUC (train-test)
sequence features	train	0.973	0.938	0.923	0.930	0.913	0.995	0.37	0.29
	test	0.901	0.556	0.556	0.556	0.500	0.704		
network features	train	0.815	0.094	0.667	0.164	0.200	0.721	–0.04	0.12
	test	0.901	0.111	1.000	0.200	0.316	0.597		
sequence and network features	train	0.949	0.813	0.912	0.860	0.830	0.976	0.19	0.17
	test	**0.926**	**0.667**	**0.667**	**0.667**	**0.625**	**0.809**		

While representation learning improves predictive power, the resulting embeddings have been perceived as black-box representations. To address this interpretability concern, we quantified the biological information captured by PDI-Spot by calculating Spearman correlation coefficients between learned embeddings and a set of 22 conventional handcrafted descriptors (Figure 3C). Our analysis reveals that sequence-derived embeddings effectively encode fundamental structural properties. Specifically, several dimensions showed correspondence with relASA and local backbone organization (secondary structure). Among the secondary structure categories, helix and turn showed comparatively high correlation with the learned embeddings, which is expected and biologically interpretable because helix, especially alpha, is the most common protein structural element used for base recognition and has compatible structural geometry to insert directly into the major groove of DNA.^57,58^ The turn connecting two helices determines the precise angular orientation of the recognition helix.^58^ The sequence embeddings appear to implicitly encode this local backbone organization, consistent with the known role of secondary structure in mediating contacts at PDIs. Additionally, these embeddings captured essential determinants of interface stability, including hydropathy, hydrogen-bond-related features, evolutionary conservation, and amino acid propensity score. Although propensity score is not highly correlated with all 13 selected embeddings, a few embeddings (sequence embedding 1042 and non/hotspot propensity and sequence embedding 30 and nonhotspot propensity) show a weak positive correlation. This partial correlation suggests that propensity score, as a global amino acid-level metric, cannot fully reflect all learned embeddings encoding position-specific and context-dependent information. Conversely, network-derived embeddings exhibited minimal correlation with traditional descriptors. This divergence is expected because graph-based representations encode topological features that emerge from the global structure of the AAN, rather than from the physicochemical properties of individual residues. Together, these results demonstrate that our model does not merely memorize data but learns biologically meaningful features, while the network embeddings provide orthogonal information that complements traditional structural descriptors.

### Validation of PDI-Spot through Representative Case Studies

To visualize the predictive accuracy of our PDI-Spot model, we used case studies on four distinct protein–DNA complexes: the RecQ catalytic core from *Cronobacter sakazakii* (PDB ID: 4TMU), LIN54, a component of the DREAM complex from *Homo sapiens* (PDB ID: 5FD3), HTH-type transcriptional repressor PurR from *Escherichia coli* (PDB ID: 1PNR), and DNA processing protein A (DprA) from *Helicobacter pylori* (PDB ID: 4LJR). In the RecQ complex, the interface contains one experimentally validated hotspot (R246-ΔΔ*G* ≥ 2 kcal/mol) and eight nonhotspots (S97, E124, R125, R275, T293, R315, W347, and R446), which is also a case study in the ESPDHot method. As shown in Figure 4A, PDI-Spot accurately identified the critical R246 residue while correctly classifying all surrounding nonhotspots, despite the high density of arginine residues at this interface, which often complicates traditional prediction. Similarly, for the human LIN54 complex, our model successfully identified both tyrosine hotspots (Y536 and Y610-ΔΔ*G* ≥ 2 kcal/mol) that are essential for DNA binding (Figure 4B). Furthermore, the lysine residue crucial for determining DNA-binding specificity and affinity in the transcriptional repressor PurR was accurately predicted as a hotspot (K55-ΔΔ*G* ≥ 2 kcal/mol) (Figure 4C). Finally, we evaluated DNA processing protein A, another case study used in the ESPDHot method, with an interface consisting of two hotspots (Y108 and F140-ΔΔ*G* ≥ 2 kcal/mol) and two nonhotspots (R52 and R143). PDI-Spot correctly identified both hotspots and nonhotspots, whereas ESPDHot inaccurately predicted R143 as a hotspot (Figure 4D). Although arginine residues are highly prone to acting as hotspots, which conventional computational methods often struggle with, PDI-Spot was able to classify R143 as a nonhotspot. These case studies demonstrate that PDI-Spot can distinguish between residues with high physicochemical similarity, such as the various arginines in RecQ, to pinpoint the exact energetic anchors of the interaction (see Figure S6 and Table S10 for an additional case study of one protein bound to two different DNA sequences). The consistent accuracy across these diverse complexes, representing both bacterial catalytic cores and human transcriptional regulators, further validates the generalizability of our representation-learning approach.

**Figure 4:**
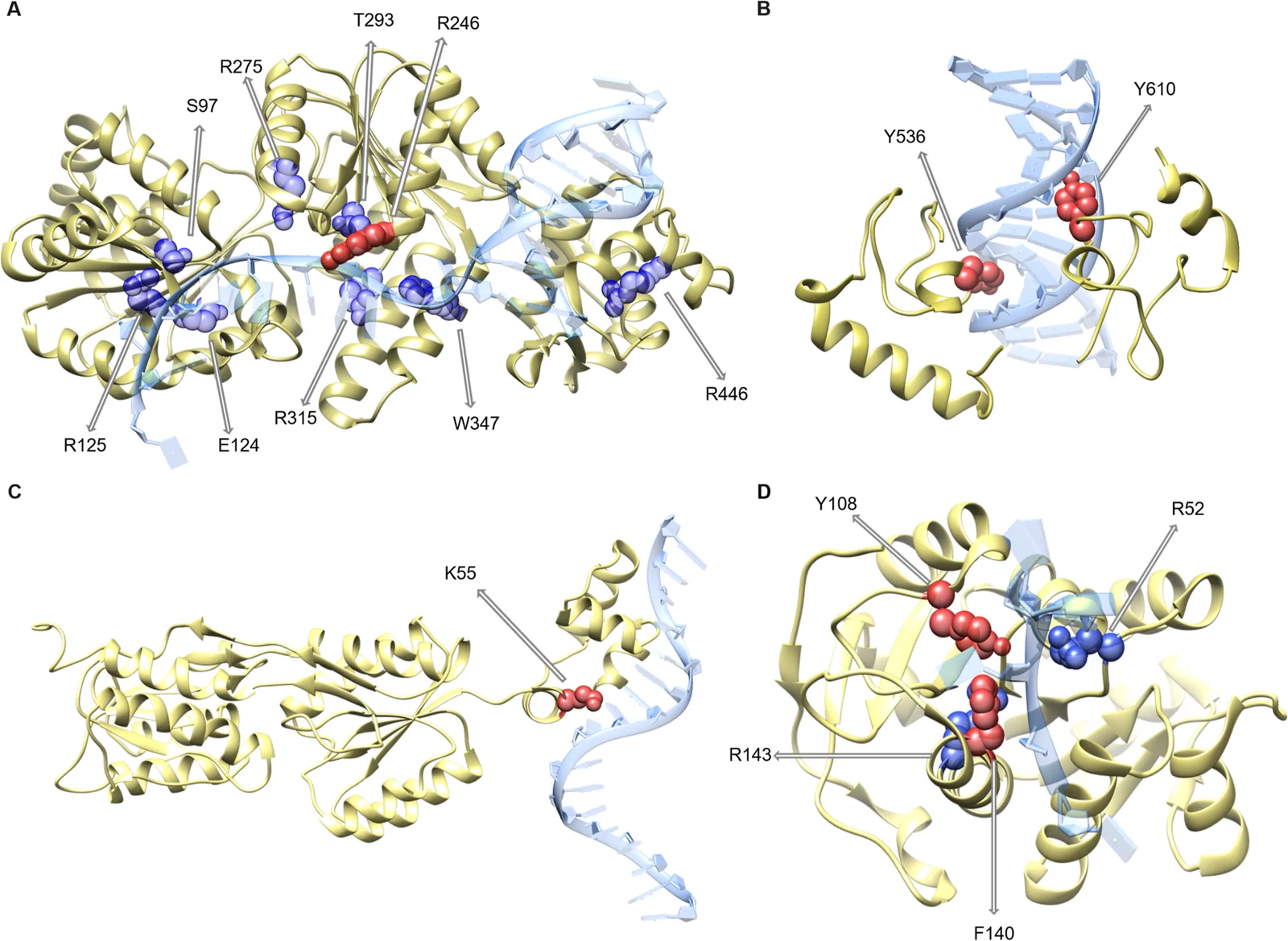
Prediction results of PDI-Spot at protein–DNA complexes 4TMU (A), 5FD3 (B), 1PNR (C), and 4LJR (D). The predicted residues are shown in spheres, with red ones being the accurately predicted hotspots and blue ones being the accurately predicted nonhotspots.

## Discussion

In this work, we introduced PDI-Spot, a novel hotspot prediction framework that exclusively utilizes protein embedding features, challenging the conventional dependence on manually calculated handcrafted features. By synthesizing the evolutionary semantics of ESM–DBP with the topological information from Node2Vec, we have shown that a residue’s properties as a hotspot are encoded in its latent biological context and its strategic position within AANs. To our knowledge, PDI-Spot is the first method to rely solely on embedding-based representations, effectively eliminating the need for traditional features. Our model utilizes a subset of 13 features from high-dimensional complex features and outperforms existing state-of-the-art methods (AUROC = 0.809, F1-score = 0.667, MCC = 0.625). It was also validated on case studies, demonstrating high precision in distinguishing hotspot from non-hotspot residues at PDIs. These results indicate that embedding features capture informative patterns relevant to hotspot characterization and can serve as an effective alternative to traditional features. The proposed PDI-Spot framework provides a simplified yet robust method for hotspot prediction.

The strength of PDI-Spot lies in its ability to bypass the noise and incompleteness inherent in manual structural annotations. By leveraging deep learning models trained on large-scale protein data, we have developed a tool that is both robust and simplified, requiring no prior expert knowledge to extract handcrafted features. On the other hand, this transition to deep embeddings opens new frontiers. “Black-box” nature of latent features remains a hurdle for direct biological interpretation, alongside the high computational cost of deep learning models. Furthermore, our framework focuses solely on the protein’s structural and evolutionary profile. Importantly, this design also offers flexibility: PDI-Spot does not require DNA coordinates as input, relying solely on protein sequence and structure. Given that experimentally determined protein–DNA complex structures remain scarce in the PDB,^59,60^ this independence from DNA structural data substantially broadens the applicability of the framework, enabling hotspot prediction for transcription factors and other DBPs where only protein structures are available. Yet, incorporating the DNA’s own conformational architecture and sequence motifs will provide a more holistic and bidirectional model, potentially elevating predictive performance even further.

PDI-Spot, trained on a thermodynamic data set with a threshold of kcal/mol, accurately predicted hotspots across diverse protein–DNA complexes from different species such as bacteria and humans. However, a limitation regarding the nature of the training data must be acknowledged. The model may not perform with the same efficacy on functional alanine-scanning mutagenesis data sets, where the definition of hotspots is based on functional assays instead of the change in binding affinity. This reduced transferability points to the need for more training data; yet, curated experimental data sets remain limited in the field. Addressing this data limitation and expanding curated databases represent critical directions for future model optimization.

Ultimately, PDI-Spot, which circumvents the manual calculation of hand-crafted features, is computationally efficient, enabling us to adapt it for broader structural bioinformatics applications. Prospectively, hotspot identification can be synergistically integrated with interface prediction and validation. It is well-known that hotspots play a significant role in interactions as energetic anchors, for which reason computationally docked structures can be scored and filtered by leveraging hotspot locations. Thus, combining hotspot identification with models that predict protein interfaces via learned representations, such as ProInterVal,^61^ can yield computationally assembled or verified molecular complexes. Such a combined framework can be readily deployed in large-scale applications. This scalability presents a significant opportunity, particularly for mapping critical interactions across entire interactomes, resulting in comprehensive structural annotations.^62^

## Supplementary Material



## Data Availability

The data sets used for model training and testing, along with the Python scripts and the model developed in this work, have been deposited in the GitHub repository at https://github.com/ebsrn0/pdi-spot.git. Additional supporting data are also provided in the Supporting Information.

## Notes

A flowchart detailing the data set preprocessing steps, a sequence similarity heatmap of the filtered proteins, flexibility profiles comparing hotspot and nonhotspot residues, visual prediction results of PDI-Spot on the PARP1-DNA complex, PDB IDs for the training and test sets, predictive performance metrics, a comprehensive performance comparison with existing methods, and residue accessibility and conservation status for PARP1 (PDF)
