# Supplementary material for: PDI-Spot: A Robust Representation Learning Framework for Identifying Energetic Hotspots in Protein–DNA Complexes
Source: J Chem Inf Model. 2026 Oct 14;66(20):13641–51. doi: 10.1021/acs.jcim.6c01579 (PMC13715517; doi:10.1021/acs.jcim.6c01579)
Supplement: Supplementary file 1 [file ci6c01579_si_001.pdf]

Supporting Information

-

PDI-Spot: A Robust Representation Learning Framework for Identifying Energetic Hotspots in Protein-DNA Complexes

Esra Basaran[1], Nurcan Tuncbag[2,3,*], Attila Gursoy[4], Ozlem Keskin[2]

[1] Computational Science and Engineering Graduate Program, Koc University, Istanbul 34450, Turkey
[2] Department of Chemical and Biological Engineering, College of Engineering, Koç University, Istanbul, Turkey
[3] School of Medicine, Koç University, Istanbul, Turkey
[4] Department of Computer Engineering, Koç University, Istanbul, 34450, Turkey

[*] Corresponding Author: Nurcan Tuncbag, Department of Chemical and Biological Engineering, College of Engineering, Koç University, Istanbul, Turkey. E-mail address: ntuncbag@ku.edu.tr

Table of Contents

Figure S1 - Flowchart of dataset preprocessing

Figure S2 - Sequence similarity heatmap of proteins

Figure S3 - Distribution of hotspot and non-hotspot residue mutations across complexes in the training dataset

Figure S4 - Structural and evolutionary analysis of the dataset

Figure S5 - Permutation tests assessing model performance and learning ability

Figure S6 - Prediction results of PDI-Spot on PARP1 bound to two different DNA sequences

Table S1 - List of PDB IDs used in this study

Table S2 - Protein structures divided into training and independent test sets

Table S3 - The hyperparameters of Node2vec

Table S4 - The hyperparameters of the final neural network model

Table S5 - The performance of feature selection methods on the training set

Table S6 - 95% confidence intervals for the performance of feature selection methods

Table S7 - Comprehensive performance comparison with other methods

Table S8 - The performance of PDI-Spot trained on ESPDHot's training set

Table S9 - The performance of the simpler baselines with different feature subsets

Table S10 - Residue accessibility and conservation status for PARP1

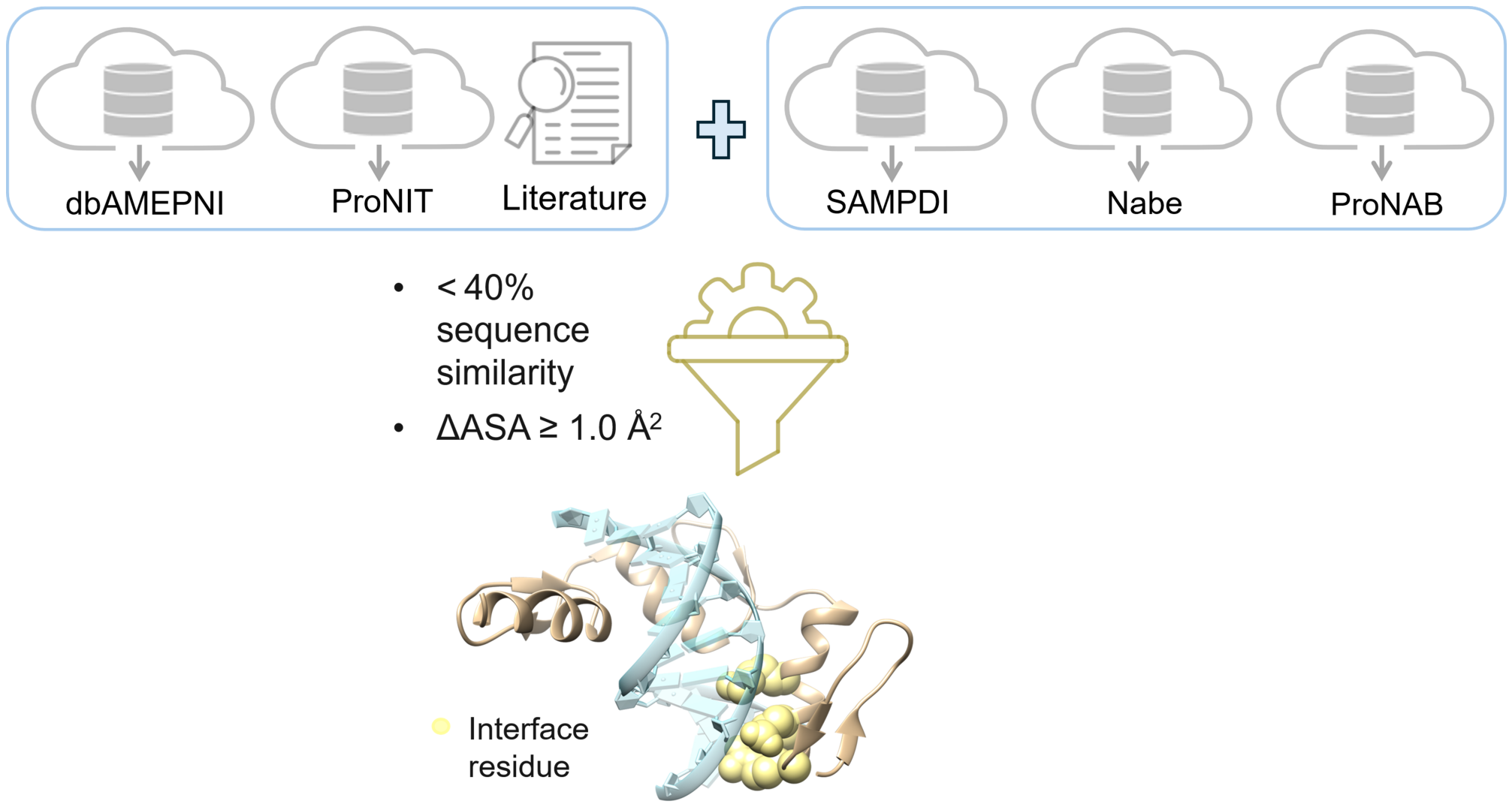

Figure S1: Flowchart of dataset preprocessing. First, six data sources were combined to build a larger dataset. Two main filters were applied to avoid redundancy in the dataset: <40% sequence similarity and a ΔASA $\geq$ 1.0 Å^2.

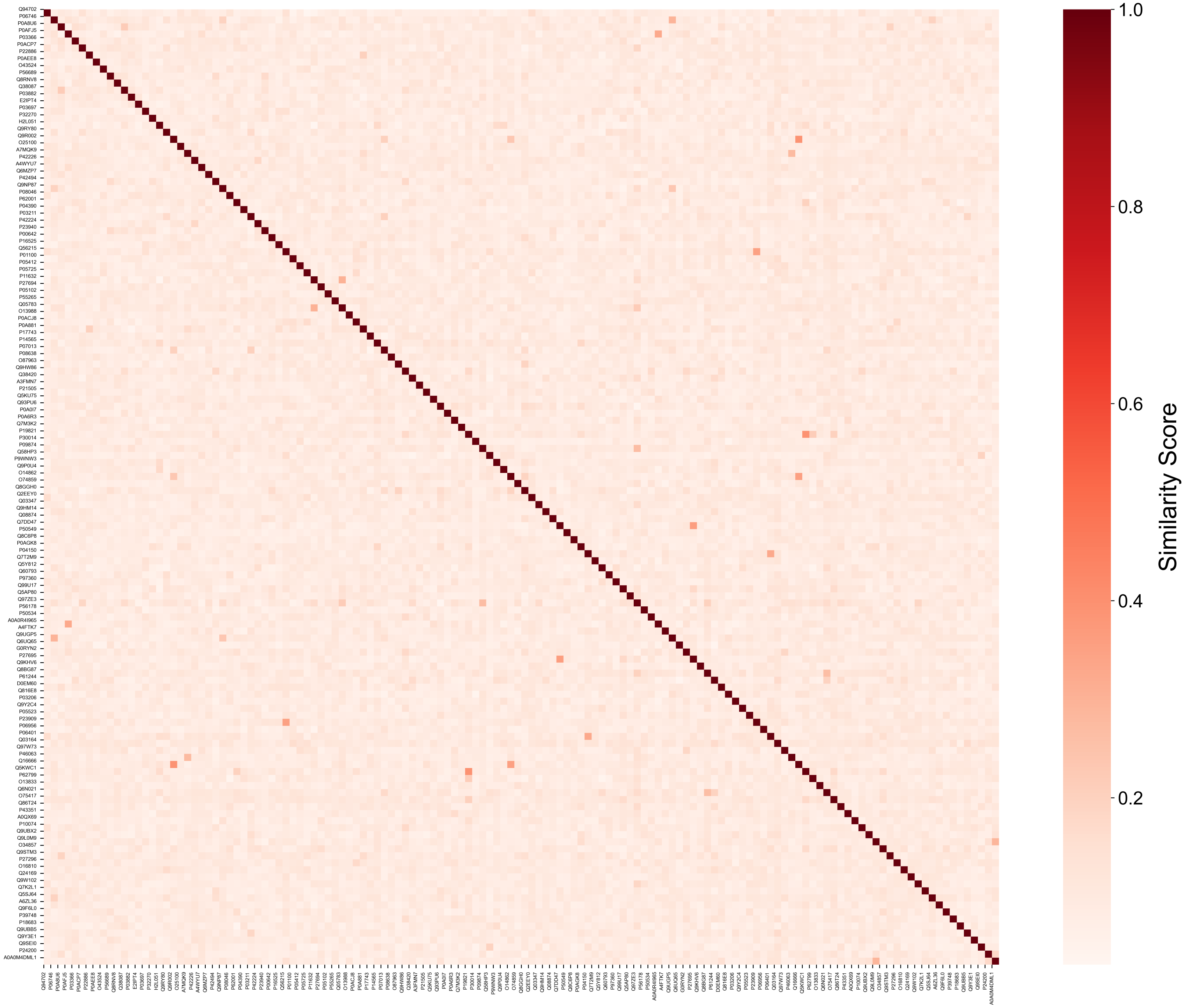

Figure S2: Sequence similarity heatmap of proteins in the training and test sets after filtering based on sequence similarity. Similarity scores are shown, with darker red indicating higher similarity (maximum = 1).

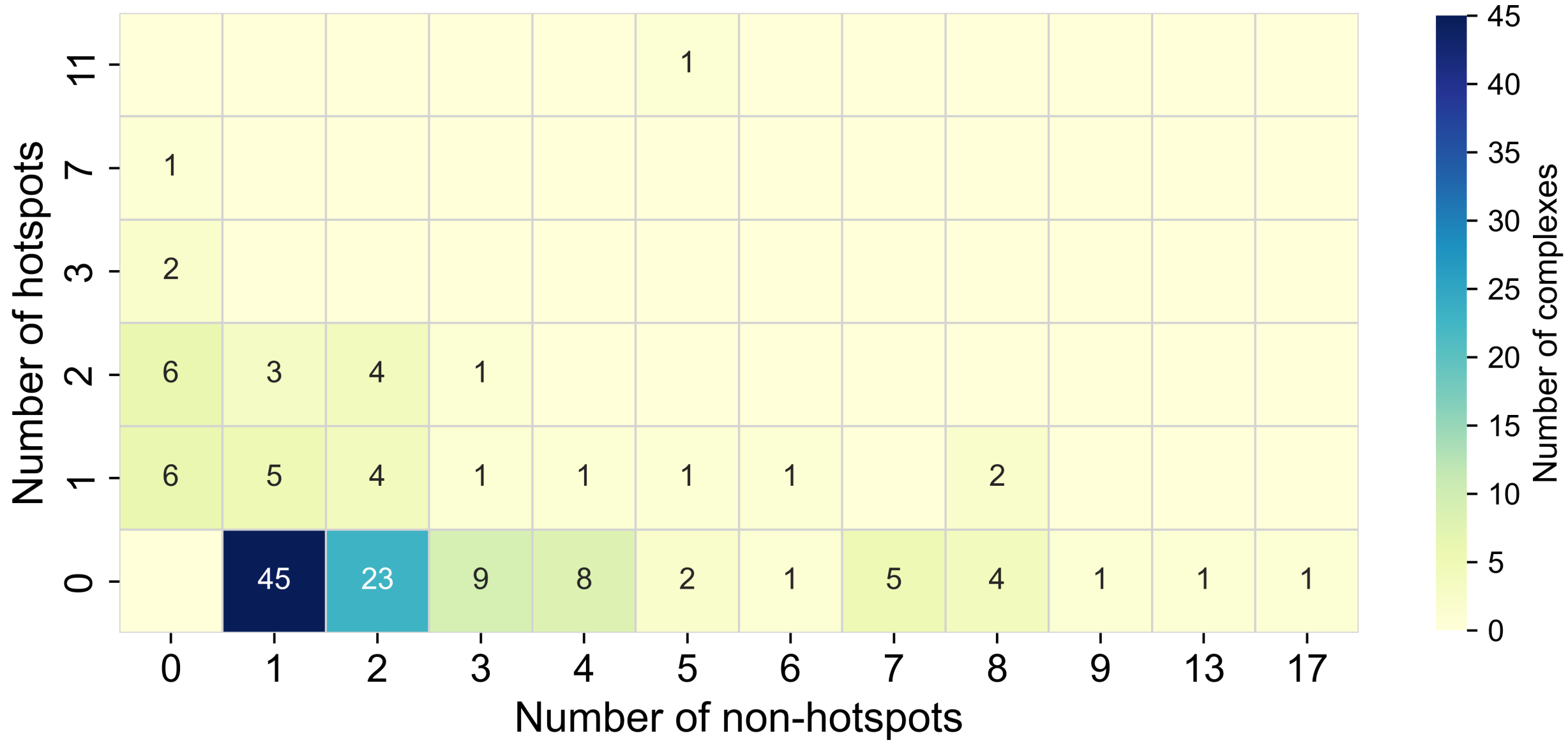

Figure S3: The number of complexes categorized by hot spot and non-hot spot residue mutations in the training dataset.

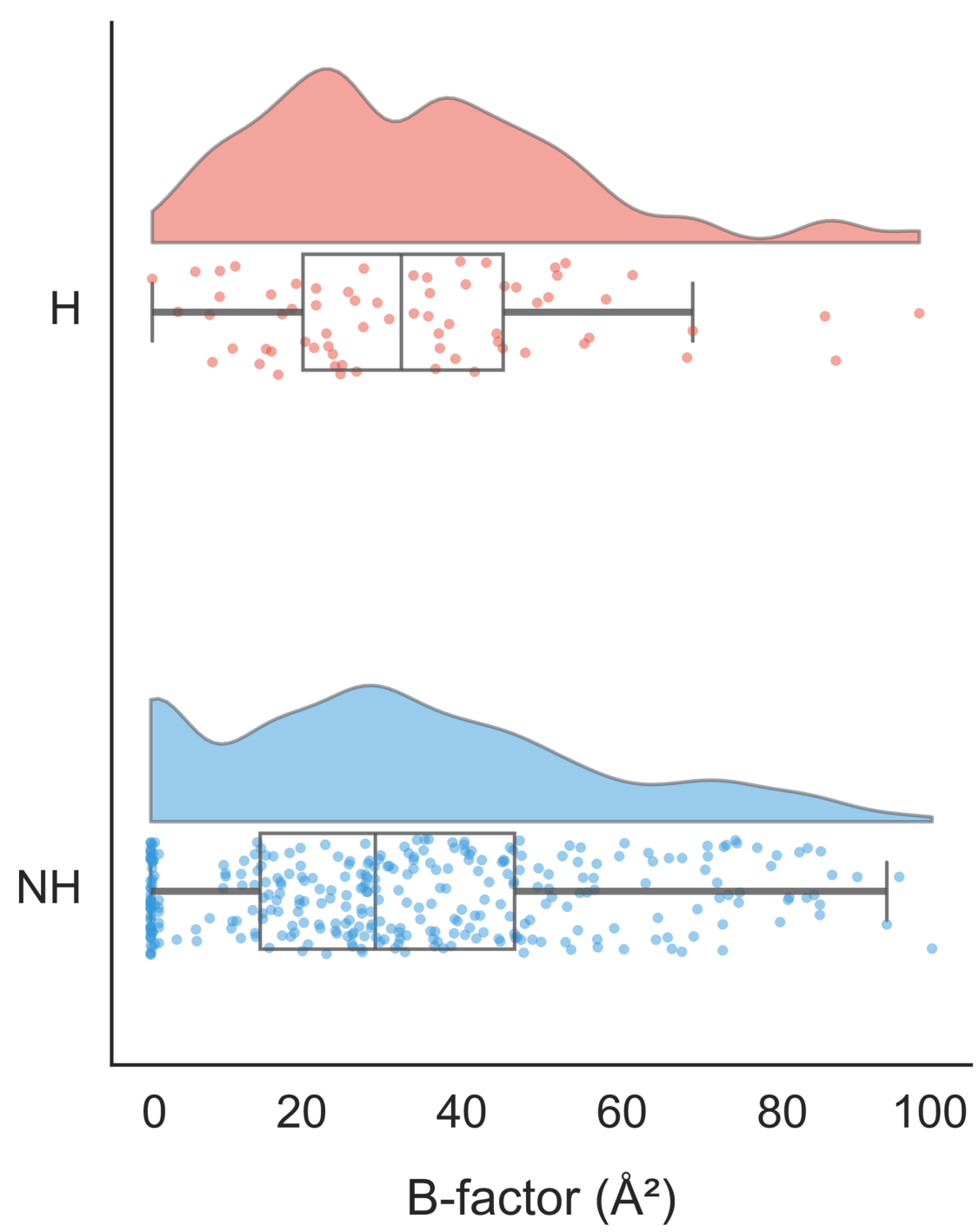

Figure S4: Flexibility profiles of hotspot (H) and non-hotspot (NH) residues based on raw Cα B-factors. Upper outliers were trimmed according to Tukey's rule ($Q3 + 1.5 \times IQR$) for visualization clarity.

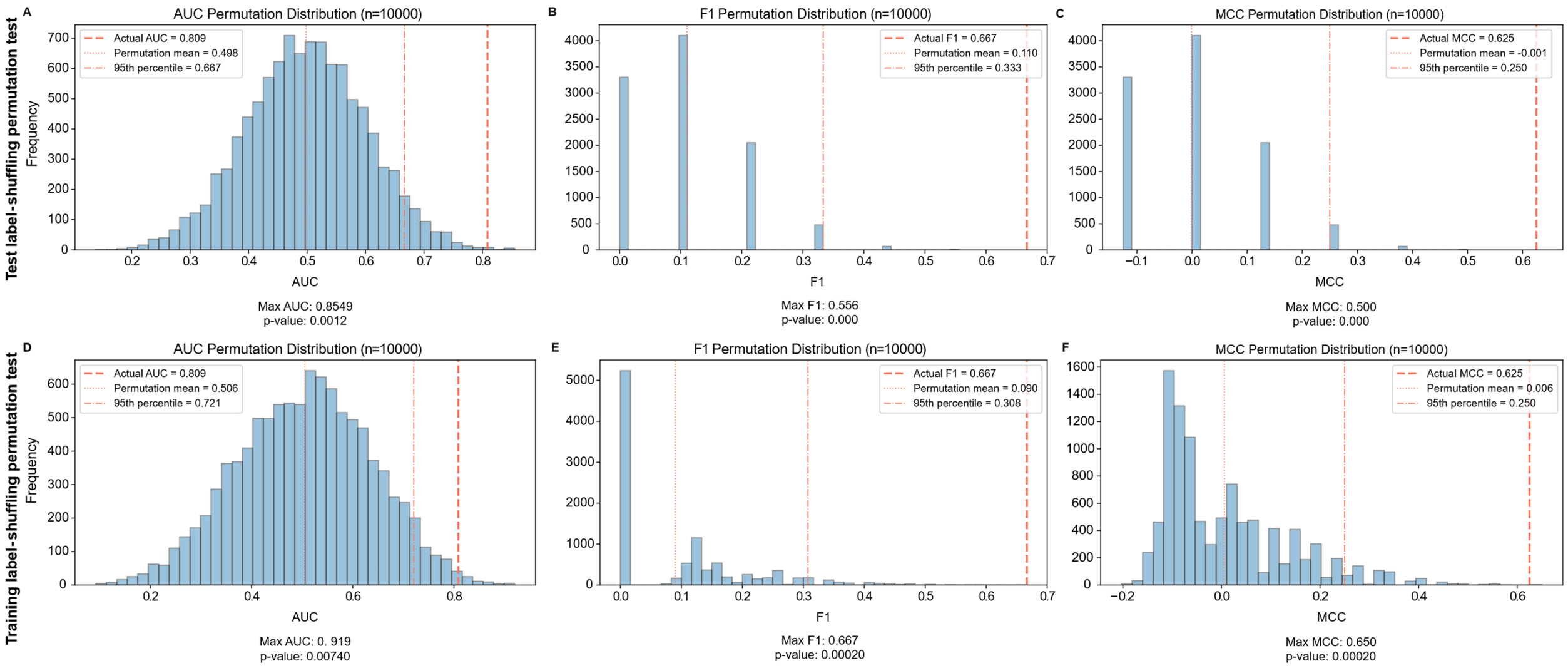

Figure S5: Permutation tests were applied to assess model performance and model's learning ability. (A–C) Test label–shuffling permutation test (n = 10,000). Class labels in the test set were randomly shuffled at each iteration with training labels kept fixed. Histogram plots show the distributions of AUC (A), F1 score (B) and MCC (C). The results indicate that the observed performance is significantly higher than expected by chance. (D–F) Training label–shuffling permutation test (n = 10,000). Labels were shuffled before training, generating a new randomized training set at each iteration and retraining the model on these label-shuffled sets. The results of the model's performance on test set show the distributions of AUC (D), F1 score (E), and MCC (F). The vertical dashed red line indicates the actual model performance, while dotted lines denote the permutation mean and the 95th percentile threshold. This highlights that our model captures meaningful biological patterns while training.

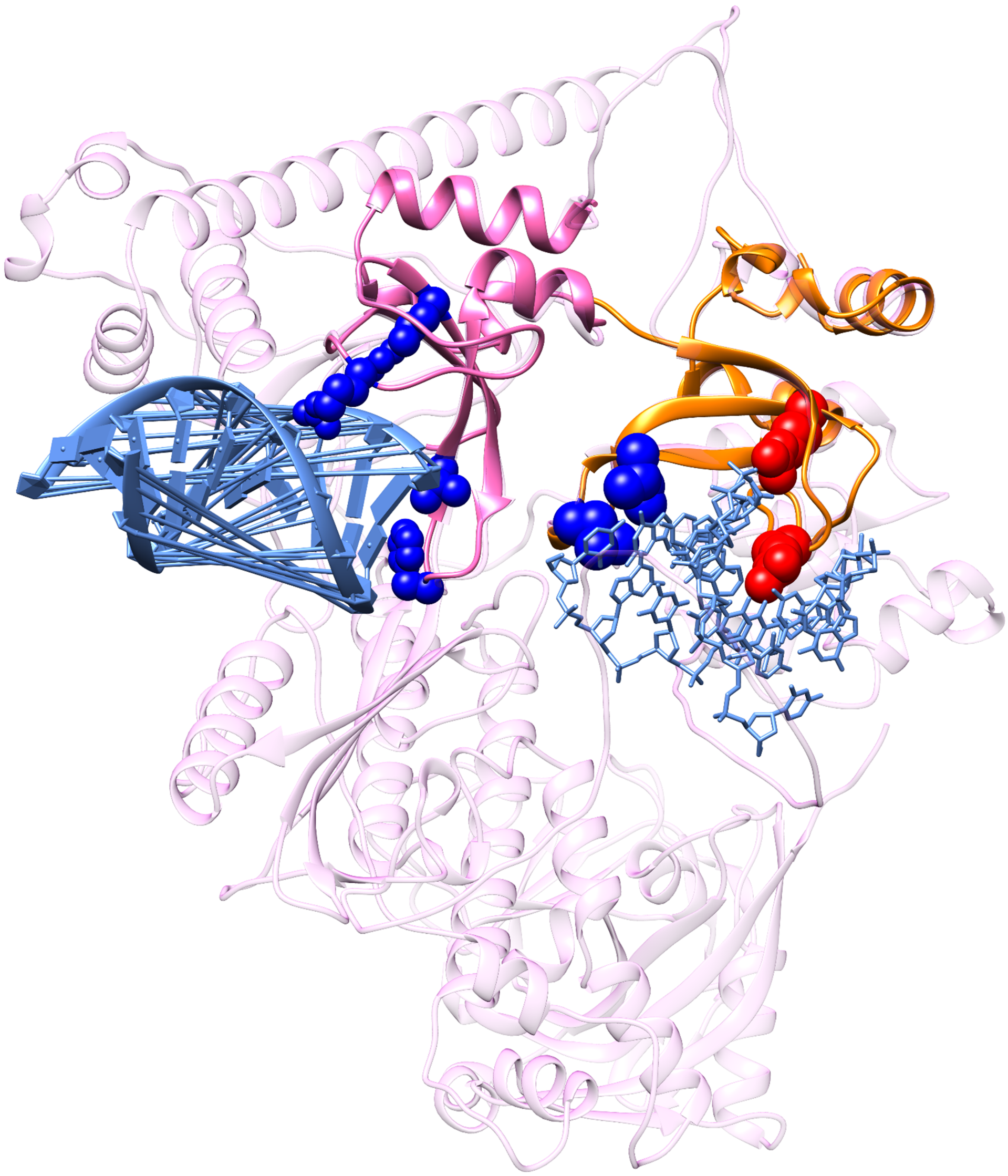

Figure S6: Prediction results of PDI-Spot on a protein-DNA complex in which PARP1 protein binding to two different DNA sequences. PARP1 contains ZF1 and ZF2 DNA-binding domains [1]. PARP1 AlphaFold structure is colored in light pink, with the ZF1 domain in pink (PDB:3OD8) and the ZF2 domain in orange (PDB:3ODC). The predicted residues are shown in spheres, with red ones being the accurately predicted hot spots and blue ones being the accurately predicted non-hot spots.

Table S1: List of PDB IDs used in this study, along with the number of hotspot (H) and non-hotspot (NH) mutations at the protein–DNA interface and the total mutation count (MC) for each structure.

**PDB**	**H**	**NH**	**MC**	**PDB**	**H**	**NH**	**MC**	**PDB**	**H**	**NH**	**MC**	**PDB**	**H**	**NH**	**MC**
1A73	1	0	1	2ERE	0	4	4	3SM4	0	4	4	4XQ8	0	1	1
1AAY	2	2	4	2FQZ	0	3	3	3SPD	0	2	2	4XR0	1	0	1
1AIS	0	2	2	2G1P	0	7	7	3UFD	1	2	3	4Y5W	0	2	2
1AZ0	0	2	2	2KKF	0	8	8	3UPU	0	2	2	4ZSF	1	0	1
1B3T	3	0	3	2MNA	0	7	7	3WPC	3	0	3	5AKM	0	1	1
1B94	2	0	2	2MXF	0	7	7	3WTS	0	1	1	5AWH	1	0	1
1BF5	0	1	1	2NTZ	0	3	3	4A15	2	0	2	5CO8	0	2	2
1BHM	0	1	1	2UZK	0	1	1	4ATK	0	2	2	5DFF	0	1	1
1BPX	0	3	3	2VLA	0	3	3	4B5F	0	2	2	5DWB	0	5	5
1CKQ	0	2	2	2VS7	0	2	2	4BNC	2	2	4	5EXH	1	3	4
1CMA	0	1	1	2VWJ	0	3	3	4CH1	1	8	9	5EYO	0	1	1
1ECR	0	3	3	2VYE	2	0	2	4DS5	0	1	1	5F55	0	7	7
1EWQ	0	1	1	2WWY	0	1	1	4GZN	0	1	1	5FD3	2	0	2
1FOS	0	9	9	2XRO	0	1	1	4HF1	0	5	5	5HRI	0	2	2
1G9Z	2	0	2	3AU6	0	1	1	4HN5	0	1	1	5KUB	0	2	2
1GXP	0	2	2	3BS1	2	1	3	4HQB	0	1	1	5SZX	0	1	1
1IO4	0	1	1	3IMB	0	2	2	4KMF	0	2	2	5T5C	0	1	1
1J5N	0	17	17	3IV5	1	1	2	4L5S	0	4	4	5TWP	0	2	2
1JMC	1	5	6	3K4X	0	1	1	4LD9	0	1	1	5VMW	0	2	2
1K82	1	1	2	3KDE	0	4	4	4LJR	2	2	4	5XRZ	0	1	1
1MHT	1	2	3	3KTQ	0	1	1	4LLN	0	2	2	5YEJ	1	1	2
1N6Q	0	1	1	3MQ6	0	1	1	4M9E	0	1	1	5YJ3	1	4	5
1OH6	0	1	1	3NCI	0	2	2	4MHG	0	1	1	5Z6Z	0	8	8
1OUQ	0	1	1	3NGZ	0	4	4	4NKU	0	1	1	5ZMO	0	2	2
1PNR	1	0	1	3NH0	0	1	1	4NM6	0	2	2	5ZUX	0	7	7
1QBJ	0	1	1	3OD8	0	4	4	4QJU	0	3	3	6A57	1	6	7
1QRV	0	3	3	3ODC	2	2	4	4QTJ	0	8	8	6ALG	2	1	3
1QZG	2	3	5	3OOL	0	1	1	4R56	0	4	4	6C1A	0	1	1
1RUN	1	1	2	3OSG	7	0	7	4RDU	0	1	1	6FWR	0	2	2
1TN9	0	13	13	3PVV	1	0	1	4RKG	1	1	2	6IIQ	0	2	2
1TRO	0	1	1	3Q8L	2	0	2	4RKH	0	1	1	6KKS	0	1	1
1TW8	0	1	1	3QFQ	0	1	1	4S04	0	3	3	6R64	1	2	3
2A0I	11	5	16	3QMG	2	1	3	4TMU	1	8	9	7CC9	1	2	3
2C7A	0	1	1	3RN2	0	8	8	4WCG	0	1	1	7JK5	0	6	6
2CCZ	0	4	4	3RNU	0	1	1	4X0Q	0	1	1				

Table S2: Protein structures divided into training and independent test sets.

Set	PDB IDs
**Test**	1A73, 1BPX, 1CMA, 1GXP, 1N6Q, 1PNR, 1TN9, 2G1P, 2UZK, 2VWJ, 3IMB, 3NCI, 3OOL, 3QFQ, 3SM4, 3UPU, 4CH1, 4HQB, 4L5S, 4LJR, 4TMU, 4Y5W, 5AWH, 5FD3, 5HRI, 5TWP
**Training**	1AAY, 1AIS, 1AZ0, 1B3T, 1BF5, 1BHM, 1CKQ, 1ECR, 1EWQ, 1FOS, 1G9Z, 1J5N, 1JMC, 1MHT, 1QBJ, 1QRV, 1QZG, 1RUN, 1TRO, 1TW8, 2A0I, 2CCZ, 2ERE, 2FQZ, 2MXF, 2NTZ, 2VLA, 2VS7, 2VYE, 2XRO, 3BS1, 3IV5, 3KDE, 3KTQ, 3NH0, 3OD8, 3ODC, 3OSG, 3PVV, 3QMG, 3RN2, 3SPD, 3UFD, 3WPC, 3WTS, 4A15, 4ATK, 4B5F, 4BNC, 4GZN, 4HF1, 4HN5, 4KMF, 4LLN, 4M9E, 4MHG, 4QJU, 4QTJ, 4R56, 4RDU, 4RKG, 4S04, 4WCG, 4XQ8, 4ZSF, 5CO8, 5DFF, 5DWB, 5EXH, 5EYO, 5F55, 5KUB, 5SZX, 5T5C, 1B94, 1IO4, 1K82, 1OH6, 1OUQ, 2C7A, 2KKF, 2MNA, 2WWY, 3NGZ, 3RNU, 4DS5, 4LD9, 4NKU, 4NM6, 4RKH, 4X0Q, 4XR0, 5AKM, 5VMW, 5XRZ, 5YEJ, 5YJ3, 5Z6Z, 5ZMO, 5ZUX, 6A57, 6FWR, 7JK5, 3AU6, 3K4X, 3MQ6, 3Q8L, 6ALG, 6C1A, 6IIQ, 6KKS, 6R64, 7CC9

Table S3: The hyperparameters of Node2vec.

Hyperparameters	Values
Embedding dimensions	64
Walk length	32
Number of walks per node	200
Return parameter (p)	0.5
In-out parameter (q)	2
Workers	4

Table S4: The hyperparameters of the final neural network model.

Hyperparameters	Values
Number of Layers	4
Hidden Size	151
Activation Function	ReLU
Learning Rate	1.89×10^{-3}
Weight Decay	9.19×10^{-5}
Dropout Probability	0.12
Batch Normalization	True

Table S5: The performance of feature selection methods on the training set.

**Feature selection methods**	**ACC**	**SEN**	**PRE**	**F1**	**MCC**
mRMR	0.83 ± 0.05	0.40 ± 0.27	0.69 ± 0.23	0.44 ± 0.21	0.40 ± 0.14
MLP	0.85 ± 0.05	0.52 ± 0.10	0.68 ± 0.14	0.57 ± 0.05	0.50 ± 0.06
Intersection of mRMR and MLP-selected features	0.83 ± 0.05	0.42 ± 0.15	0.58 ± 0.13	0.46 ± 0.12	0.38 ± 0.11
Sequential feature selection: mRMR → MLP	0.88 ± 0.04	0.76 ± 0.13	0.67 ± 0.07	0.71 ± 0.04	0.64 ± 0.07
RFE	0.81 ± 0.07	0.12 ± 0.18	0.21 ± 0.29	0.14 ± 0.20	0.11 ± 0.15
Elastic Net	0.92 ± 0.05	0.79 ± 0.15	0.80 ± 0.16	0.79 ± 0.15	0.74 ± 0.17

Table S6: 95% confidence intervals for the performance of feature selection methods on the test set.

**Feature selection methods**	**95% CI - ACC**	**95% CI - SEN**	**95% CI - PRE**	**95% CI - F1**	**95% CI - MCC**
mRMR	[0.836–0.966]	[0.448–0.664]	[0.448–0.664]	[0.448–0.664]	[0.391–0.609]
MLP	[0.789–0.939]	[0.448–0.664]	[0.310–0.524]	[0.367–0.585]	[0.299–0.513]
Intersection of mRMR and MLP-selected features	[0.869–0.983]	[0.564–0.770]	[0.564–0.770]	[0.564–0.770]	[0.520–0.730]
Sequential feature selection: mRMR → MLP	[0.789–0.939]	[0.448–0.664]	[0.310–0.524]	[0.367–0.585]	[0.299–0.513]
RFE	[0.805–0.949]	[0.448–0.664]	[0.347–0.563]	[0.391–0.609]	[0.325–0.541]
Elastic Net	[0.836–0.966]	[0.043–0.179]	[1.000–1.000]	[0.113–0.287]	[0.215–0.417]

Table S7: The comprehensive performance comparison of our method with other methods on the test set. Reported values for existing methods are sourced from the study by Tao et al. [2].

**Methods**	**ΔΔG**	**ACC**	**SEN**	**SPE**	**PRE**	**F1**	**MCC**
PDI-Spot (our model)	2.0	**0.926**	**0.667**	**0.958**	**0.667**	**0.667**	**0.625**
ESPDHot	2.0	0.889	**0.667**	0.917	0.500	0.571	0.516
PreHots	1.0	0.704	0.552	0.788	0.593	0.571	0.346
PrPDH	1.0	0.617	0.483	0.692	0.467	0.475	0.174
premPDI	1.0	0.580	0.552	0.596	0.432	0.485	0.142
premPDI	2.0	0.802	0.222	0.875	0.182	0.200	0.089
SAMPDI_3D	1.0	0.481	0.310	0.577	0.290	0.300	-0.111
SAMPDI_3D	2.0	0.864	0.000	0.972	0.000	0.000	-0.056
mCSM_NA	1.0	0.654	0.034	**1.000**	**1.000**	0.067	0.150
mCSM_NA	2.0	0.889	0.000	**1.000**	0.000	0.000	0.000

Table S8: Performance comparison of PDI-Spot trained on ESPDHot's training set and ESPDHot on the test set.

Model	ACC	SEN	SPE	PRE	F1	MCC
PDI-Spot (trained on ESPDHot training set)	0.926	0.556	0.972	0.714	0.625	0.590
ESPDHot	0.889	0.667	0.917	0.500	0.571	0.516

Table S9: The performance of the simpler baselines with different feature subsets.

Model	ACC	SEN	PRE	F1	MCC	AUROC
LR (selected 13 features)	0.728	0.666	0.24	0.353	0.274	0.776
RF (handcrafted only)	0.857	0.125	0.2	0.154	0.083	0.504
RF (ESM-DBP only)	0.901	0.111	1	0.2	0.316	0.750
RF (Node2Vec only)	0.876	0.222	0.4	0.286	0.235	0.634
RF (selected 13 features)	0.901	0.111	1	0.2	0.316	0.678
LR (11 features selected by DT)	0.667	0.555	0.178	0.270	0.156	0.617
RF (11 features selected by DT)	0.802	0.111	0.111	0.111	0	0.625
NN (11 features selected by DT)	0.827	0.333	0.273	0.3	0.204	0.711
XGBoost (raw embeddings)	0.840	0.000	0.000	0.000	-0.081	0.593
XGBoost (selected 13 features)	0.901	0.556	0.556	0.556	0.500	0.769

Table S10: Residue accessibility and conservation status for PARP1 (PDB: 3OD8 and 3ODC).

PDB	Residues	Label	Accessibility-Conservation Status
3OD8	18R	NH	Exposed - Functional
3OD8	34R	NH	Exposed - Functional
3OD8	44F	NH	Exposed
3OD8	48V	NH	Exposed
3ODC	122R	H	Exposed - Functional
3ODC	138R	H	Exposed - Functional
3ODC	151L	NH	Exposed
3ODC	154I	NH	Exposed

References

(1) Langelier, M.-F.; Planck, J. L.; Roy, S.; Pascal, J. M. Crystal Structures of Poly(ADP-ribose) Polymerase-1 (PARP-1) Zinc Fingers Bound to DNA: STRUCTURAL AND FUNCTIONAL INSIGHTS INTO DNA-DEPENDENT PARP-1 ACTIVITY*. *Journal of Biological Chemistry* **2011**, *286*, 10690–10701.

(2) Tao, L.; Zhou, T.; Wu, Z.; Hu, F.; Yang, S.; Kong, X.; Li, C. ESPDHot: An effective machine learning-based approach for predicting protein-DNA interaction hotspots. *J. Chem. Inf. Model.* **2024**, *64*, 3548–3557.
